# Exosomal miRNA Let-7 from Menstrual Blood-Derived Endometrial Stem Cells Alleviates Pulmonary Fibrosis through Regulating Mitochondrial DNA Damage

**DOI:** 10.1155/2019/4506303

**Published:** 2019-12-16

**Authors:** Lifang Sun, Min Zhu, Wei Feng, Yiping Lin, Jia Yin, Juan Jin, Yunguang Wang

**Affiliations:** ^1^Department of Tuberculosis, Hangzhou Red Cross Hospital, Hangzhou, 310003 Zhejiang, China; ^2^Department of Radiation Oncology, Institute of Cancer Research and Basic Medical Sciences of Chinese Academy of Sciences, Cancer Hospital of University of Chinese Academy of Sciences, Zhejiang Cancer Hospital, Hangzhou, 310022 Zhejiang, China; ^3^Jinhua Polytechnic, Jinhua, 321007 Zhejiang, China; ^4^Changhai Hospital, Second Military Medical University, Shanghai 200433, China; ^5^Department of Nephrology, Zhejiang Provincial People's Hospital, Zhejiang 310014, China; ^6^People's Hospital of Hangzhou Medical College, Zhejiang 310014, China; ^7^Institute of Nuclear-Agricultural Sciences, Zhejiang University, Hangzhou, 310058 Zhejiang, China

## Abstract

Idiopathic pulmonary fibrosis (IPF) is a prototype of chronic, progressive, and fibrotic lung disease with high morbidity and high mortality. Menstrual blood-derived stem cells (MenSCs) have proven to be an attractive tool for the treatment of acute lung injury and fibrosis-related diseases through immunosuppression and antifibrosis. However, whether MenSC-derived exosomes have the similar function on pulmonary fibrosis remains unclear. In the present study, exosomes secreted from MenSCs (MenSCs-Exo) were verified by transmission electron microscope (TEM), nanoparticle tracking analyzer (NTA), and western blotting. And MenSC-Exo addition significantly improved BLM-induced lung fibrosis and alveolar epithelial cell damage in mice, mainly reflected in BLM-mediated enhancement of the fibrosis score, blue collagen deposition, dry/wet gravity ratio, hydroxyproline and malondialdehyde levels, and downregulation of glutathione peroxidase, which were all robustly reversed by MenSC-Exo management. Additionally, BLM- and TGF-*β*1-evoked cellular reactive oxygen species (ROS), mitochondrial DNA (mtDNA) damage, and cell apoptosis were rescued by MenSCs-Exo *in vivo* and *in vitro*. Further study indicated that the MenSCs-Exo could transport miRNA Let-7 into recipient alveolar epithelial cells. Let-7 inhibitor administration significantly blocked the exosome-mediated improvement role on lung fibrosis in mice. Mechanistically, Let-7 was able to regulate the expression of lectin-like oxidized low-density lipoprotein receptor-1 (LOX1) through binding to its 3′-UTR region. Forced expression of LOX1 promoted the expression of apoptosis-related protein and mtDNA damage markers via regulating NLRP3 which was also confirmed in BLM model mice under the combination therapy of the exosome and Let-7 inhibitor. Collectively, this study demonstrates that exosomal Let-7 from MenSCs remits pulmonary fibrosis through regulating ROS, mtDNA damage, and NLRP3 inflammasome activation. This provides a new approach of exocytosis on the treatment of fibrotic lung disease.

## 1. Introduction

Idiopathic pulmonary fibrosis (IPF) is a chronic, fatal interstitial lung disease that usually manifests as interstitial pneumonia in radiology or histology with a poor prognosis [1, 2]. According to the statistics, the mortality rate of IPF is estimated to be 2.54 to 11.08 per 100,000 people which is increasing with age [3]. IPF primarily affects individuals between the ages of 60 and 75 and exhibits a highly variable disease process [4]. Traditionally, corticosteroids and/or cytotoxic agents such as prednisone are usually used to cure IPF disease [5]. Lung transplantation combined with drugs such as nintedanib and pirfenidone is also used to alleviate the progression of IPF. However, these therapeutic strategies are not effective for the treatment of fibrotic process, and more exploration to address this issue is needed.

According to the previous studies, the pathogenesis of pulmonary fibrosis involves impaired alveolar epithelial cells [6], macrophage activation [7], inflammatory cell aggregation and activation [8], fibroblast proliferation and collagen production, fas-mediated apoptosis, and many other factors [9]. Accumulating evidence suggests that abnormal mitochondrial reactive oxygen species (ROS) induces mitochondrial DNA (mtDNA) damage during the procedure of pulmonary fibrosis. ROS are involved in the pathogenesis of aging and lung disease, including IPF and lung cancer [10]. ROS can oxidize a variety of cellular targets including DNA, proteins, and lipids that activate a wide range of biological processes such as mitochondrial dysfunction, DNA damage response, apoptosis, and signal transduction, leading to tissue damage, abnormal wound healing, and fibrosis [11–13]. Furthermore, IPF is not caused by infectious stimuli, so danger-associated molecular patterns (DAMPS) released by injured or activated cells may be more relevant to IPF in this clinical setting [14]. As a critical ligand of DAMPS, mtDNA is also released by necrotic cells [15] and living cells in response to various stressors [16]. Studies have reported that mtDNA exposure is sufficient to activate macrophages and fibroblasts in experimental settings, including experimental pulmonary fibrosis [17]. And under conditions of pulmonary fibrosis, mtDNA participates in the formation of neutrophil extracellular traps that contribute to inflammation and tissue remodeling [18]. Therefore, the acceleration of ROS and mtDNA damage are the major features of pulmonary fibrosis.

At present, stem cell regenerative therapy brings hope to the treatment of pulmonary fibrosis due to its special biological characteristics (multidirectional differentiation, immune regulation, paracrine characteristics, etc.). Human umbilical cord MSCs (hucMSCs) can reduce liver fibrosis by inhibiting EMT [19]. Mesenchymal stem cell (MSC) transplantation can reduce lung fibrosis and lung damage in a bleomycin- (BLM-) induced animal model of pulmonary fibrosis [20, 21]. Actually, stem cell-based therapies regulate inflammation and fibrosis pathways mainly through its paracrine action. It is found that amniotic epithelial-derived exosomes restrict lung injury and enhance endogenous lung repair ability, resulting in the reduction of fibrosis on day 7 of treatment [22]. The paracrine effects of stem cells are mainly attributed to exosomes secreted from stem cells, and small RNAs and proteins carried in exosomes are key substances for their repair function. Exosomes derived from MSCs are able to alleviate renal fibrosis by delivering exogenous microRNA-let7c [23]. Menstrual blood-derived endometrial stem cells (MenSCs), novel adult stem cells from human menstrual blood, have recently been evaluated as an attractive new stem cell therapy. Once injected, MenSCs mainly accumulate in the lungs and are successfully used to prevent acute lung injury [24] and to treat diseases such as cardiac fibrosis [25]. Zhao et al. [25] found that human MenSCs protect lungs from pulmonary fibrosis through its immunosuppression and antifibrosis function. Specifically, MenSCs can reduce the production of collagen fibers, the expressions of TGF-*β* and proapoptotic gene Bax, while effectively inhibiting the expression of the antiapoptotic gene Bcl-2 and antifibrotic genes HGF and MMP-9 [25]. However, the underlying mechanism of MenSCs mediating the intervention of pulmonary fibrosis remains to be further studied.

Herein, we revealed that human MenSC-derived exosomes relieved BLM-induced lung fibrosis and alveolar epithelial cell damage. Importantly, the exosomal Let-7 was the key protective factor of MenSCs which suppressed the activation of ROS and mtDNA damage through regulating NLRP3 signaling by targeting LOX1. These findings laid the foundation for the further application of MenSCs in clinical treatment.

## 2. Materials and Methods

### 2.1. Preparation and Identification of MenSCs

Approximately 5 ml of menstrual blood was collected from healthy female subjects with normal menstrual cycles. The menstrual blood was transferred to PBS containing amphotericin B (Sigma-Aldrich, US) and penicillin/streptomycin (1%) (HyClone, US). After incubation at 4°C for 24 hours, the sample was centrifuged at 1600 g for 10 minutes at 4°C, and the supernatant was subjected to microbiological examination. Mononuclear cells were separated by Ficoll-Paque (Thermo Fisher Scientific, USA) density gradient centrifugation and washed twice with PBS. Purified monocytes were cultured using Chang's medium (Laboserv, Germany). After 4-6 days of culture, cells were digested with trypsin (Boster, China) for passage. The 3rd-6th passage cells were taken to carry out the experiment. For the identification of MenSCs, the expression levels of stem cell positive markers CD44, CD90, and CD105 and negative markers CD34 and CD45 (Thermo Fisher Scientific, USA) were detected by flow cytometry (BD FACSCalibur, USA).

### 2.2. Adipogenic and Osteogenic Differentiation and Authentication of MenSCs

The prepared MenSCs in passage 3 were subjected to adipogenic differentiation induction. The cells were cultured with a fat induction solution (glucose-free DMEM (HyClone, USA), 10% FBS (Gibco, USA), 1 *μ*mol/l dexamethasone (Sigma, USA), and 0.5 mmol/l IBMX (Sigma, USA)), after treatment with 0.2 mmol/l indomethacin (Sigma, USA) and 5 *μ*g/ml insulin (Sigma, USA) for 3 days. Then, it was replaced with a fat retention solution (5 *μ*g/ml insulin, 10% FBS, and H-DMEM (HyClone, USA)) for 1 day. After the cycle was repeated 3 times, it was treated with fat retention solution for 7 days. Finally, the results of cell differentiation were identified using Oil Red O staining (Beyotime, China). For the detection of osteogenic differentiation, 1 × 10^5^ cells were plated on coverslip in a 6-well plate. After incubation for 24 h, cells were induced with osteogenic medium containing 10% FBS, 10 nM dexamethasone, 10 mM *β*-glycerophosphate, and 50 *μ*g/ml ascorbic acid in high-glucose DMEM medium. Normal high-glucose DMEM was served as the control group. At day 14, the cells were harvested for osteogenic measurement using Alizarin Red staining.

### 2.3. Isolation and Verification of MenSC-Derived Exosome

Conditioned medium was collected after culturing MenSCs for 2 days using low-sugar medium (HyClone, USA) without fetal bovine serum. Exosomes were isolated utilizing an exosome extraction kit (Wako Pure Chemicals Industry, 293-77601) by differential centrifugation according to the manufacturer's protocol. Exosomes were verified by transmission electron microscope (TEM) (Hitachi, Japan) and a nanoparticle tracking analyzer (NTA) (ZetaView, Particle Metrix, Germany) according to a previous study [26]. Exosomes were quantified using a BCA protein concentration assay (Beyotime, China) and authenticated by western blotting using primary antibodies against TSG101 (ab83, UK), CD9 (ab223052, UK), CD63 (ab216130, UK), and calnexin (ab22595, UK). The extracted exosomes are dissolved in physiological saline for animal administration.

### 2.4. Animal Model and Treatment

Animal experiments were carried out with approval by the Ethics Committee of Zhejiang University and conducted in accordance with the China Code of Practice for the Care and Use of Animals for Scientific Purposes. 6-week-old male C57BL/6J wild-type (WT) mice (SLAC, Shanghai, China) were used for the establishment of a lung fibrosis model. The mice were randomly divided into a control group, exosome group, BLM group, and exosome treatment group (*n* = 6 for each group). BLM-induced pulmonary fibrosis mice were established according to the methods of the previous literature [27]. Briefly, BLM (Nippon Kayaku, Japan) was intratracheally administered to mice by dissolving in a dose of 1.5 U/kg in 0.05 ml of sterile saline. The control group was treated with 0.05 ml of sterile saline using the same method. 21 days after model establishment, the mice was injected with exosomes (0.5 mg/kg/day) or isometric saline through the tail vein for 7 days. For the miRNA intervention experiment, exosomes (0.5 mg/kg/day) plus antagomiR-NC (10 mg/kg in 50 *μ*l saline) or exosomes (0.5 mg/kg/day) plus antagomiR-Let-7 (10 mg/kg in 50 *μ*l saline) were subsequently injected via the tail vein for a total of 7 days. AntagomiR-NC and antagomiR-Let-7 were purchased from Gima Bio, China. On the 28^th^ day after modeling, alveolar lavage fluid, venous blood serum, and lung tissue were collected from each group of mice. The ratio of dry and wet weight was calculated. Histopathology was conducted by HE staining and Masson staining. The fibrotic area and Ashcroft score were used to evaluate the degree of pulmonary fibrosis. Detections of malondialdehyde (MDA), hydroxyproline (HyP) content, and alveolar lavage fluid for oxidase (GSH-Px) were processed by using corresponding detection kits.

### 2.5. Cell Culture and Transfection

The MLE-12 cell line was purchased from the American Type Culture Collection (ATCC, Manassas, VA, US). MLE-12 cells were cultured in Dulbecco's modified Eagle's medium (DMEM) (Invitrogen, Grand Island, NY) supplemented with 1% penicillin/streptomycin and 10% (*v*/*v*) fetal bovine serum (FBS) and maintained at 37°C in a 5% CO_2_ incubator. Cells were seeded in 6-well plates or 100 mm culture dishes and grown to confluence. For cell transfection, the LOX1 cDNA fragment was inserted into the pCI-neo-LOX1 plasmid (R&D Systems). The pCI-neo-LOX1 plasmid was then transfected into MLE-12 cells by using Lipofectamine 2000 (Invitrogen) according to the manufacturer's protocol. After 48 h of transfection, TGF-*β*1 (1 ng/ml) was added for another 48 h. 100 *μ*g/ml exosomes, 100 *μ*g/ml exosomes plus NC inhibitor, or 100 *μ*g/ml exosomes plus Let-7 inhibitor was transfected into the cells. After that, the cells were collected for the next analysis.

### 2.6. Comet Experiment

The treated cells were subjected to single cell gel electrophoresis experiments according to the procedure of a DNA Damage Detection Kit (Nanjing Jiancheng, China). Briefly, the cells were resuspended to a density of 1 × 10^6^ cells/ml. The cell-containing low-melting agarose LMA was dropped onto a normal melting agarose gel. The surface is then covered with a low-melting agarose LMA. After the cells were lysed, an alkaline electrophoresis buffer was added to decompose the DNA base for single cell electrophoresis. Finally, propidium iodide (PI) staining was used to observe DNA damage in cells using a fluorescence microscope (Echo Revolve, US).

### 2.7. ROS Measurement

ROS were determined by using the 2′,7′-dichlorofluorescin diacetate (DCFH-DA) assay. In brief, MLE-12 cells and single cell suspensions (no less than 1 × 10^6^ cells) obtained from lung tissues were suspended in 1 mM DCFH-DA at 37°C for 30 min. After incubation, cells were washed twice with PBS and resuspended in a PBS buffer. Fluorescence density of ROS accumulation was measured with a flow cytometry system (BD Biosciences, San Jose, CA, USA) at the wavelength 485/530 nm.

### 2.8. Apoptosis Assay

An apoptosis assay was performed according to the manufacturer's instruction of an Annexin V/PI Apoptosis Detection kit (BD Biosciences, Franklin Lakes, NJ, USA). Briefly, the cells were resuspended at a density of 1 × 10^6^ cells/ml. And 100 *μ*l suspended cells were coincubated with Annexin V-FITC and PI staining solution in the falcon test tube for 10-15 mins in darkness. Then, a binding buffer was added and mixed on ice. The apoptotic cells were measured by flow cytometry (BD FACSCalibur, USA) within 1 h.

### 2.9. mtDNA Damage Detection

The cell mtDNA damage assay was performed according to the instruction from a mitochondrial DNA extraction kit (BioVision, US). mtDNA/18sRNA expression levels were detected by PCR (Applied Biosystems Veriti, USA). The primers for mtDNA detection were as follows: forward primer, 5′-CACCCAAGAACAGGGTTTGT-3′; reverse primer, 5′-TGGCCATGGGTATGTTGTTAA-3′. The primers for 18sRNA were as follows: forward primer, 5′-TAGAGGGACAAGTGGCCTTC-3′; reverse primer, 5′-CGCTGAGCCAGTCAGTGT-3′. The primers were synthesized by Shanghai Shenggong, China.

### 2.10. Shuttling Assays

Exosomes were labeled with 4 *μ*g/ml PKH26 dye (Sigma, USA) for 5 min, and then 3% BSA (Aladdin, China) was added to stop the staining reaction. After centrifugation using a 300 kDa ultrafiltration centrifuge tube (Sartorius Vivaspin 6, Germany), exosomes were washed three times with PBS to allow removal of unbound dye. Labeled exosomes were added to MLE-12 cells for 48 h, and the images were observed under a fluorescence microscope (control group added unlabeled exosomes). For a shuttling assay of a Cy3-labeled-miRNA precursor, MenSCs were transfected with Cy3-labeled Let-7 mimic (Guangzhou RiboBio, China). Then, exosomes secreted from MenSCs transfected with Cy3-labeled Let-7 mimic, or Cy3 alone was extracted and added to MLE-12 cells (not expressing Cy3) for 48 h. The fluorescence intensity of Cy3 in the alveolar epithelial cells was observed by fluorescence microscopy.

### 2.11. Luciferase Activity Assay

293T cells were uniformly inoculated into a 24-well plate at a density of 2 × 10^5^ cells per well and cultured in 5% CO_2_ saturated humidity at 37°C. Wild-type and mutant LOX1 3′-UTR dual luciferase plasmids were transferred into 293T cells using Lipofectamine™ 2000 (Thermo Fisher, 11668-019). After 6 hours, the mixture was aspirated and replaced with normal medium. 24 h posttransfection, the assay was performed according to the instruction of Dual-Luciferase® Dual Luciferase Reporter Detection System kits (Promega, E1910). The fluorescence intensity was measured using a Synergy H1 plate reader (BioTek, Synergy™ H1). The RLU value of sea pansy (*Renilla reniformis*) luciferase was used as an internal reference. Luciferase activity was calculated by the ratio of sample value and Renilla value.

### 2.12. qRT-PCR

Animal tissues and intracellular total RNA were extracted using the standard TRIzol method (Invitrogen, USA). It is then reversed into cDNA using the iScript cDNA synthesis kit (Bio-Rad). qPCR was performed using a mirVana™ qRT-PCR miRNA Detection Kit (Invitrogen, USA) and SYBR Premix Ex Taq™ II kit (TaKaRa, Japan). The samples were loaded in triplicate on a CFX Connect™ Real-time PCR Detection System (Applied Biosystems, Foster City, CA, USA). Data were analyzed using the Bio-Rad CFX software. GAPDH (for mRNAs) and U6 (for microRNAs) were used as internal controls for normalization. The sequences of the PCR primers are shown in Table 1.

### 2.13. Western Blot

Total protein was extracted and an equal amount of protein was electrophoresed on sodium lauryl sulfate polyacrylamide gel (SDS-PAGE). The protein was then transferred to a PVDF membrane and blocked with 5% skim milk powder for 2 h. Then, primary antibodies against LOX1 (Abeam, ab60178, 1 : 1000), NLRP3 (Abcam, ab232401, 1 : 1000), cleaved caspase 3 (Novus, MAB835, 1 : 1000), SIRT3 (Abcam, ab189860, 1 : 1000), and aconitase 2 (NOVUS, NBP1-90264, 1 : 1000) were added and incubated at 4°C for the night. GAPDH (Abcam, ab8245, 1 : 2000) was served as the internal control. Then, the samples were incubated with HRP-conjugated secondary antibody (1 : 2000) for 2 hours at room temperature. Chemiluminescence detection was performed using a SuperSignal West Pico chemiluminescent substrate.

### 2.14. Statistical Analysis

Data were presented as mean ± standard deviation (SD) and analyzed by SPSS 25.0 software (SPSS Inc., Chicago, IL, USA). One-way analysis of variance (ANOVA) was used to analyze and compare the data between the saline and BLM groups. Multiple groups with and without exosome/Let-7 inhibitor treatment were compared by two-way ANOVA using GraphPad Prism 7.0 software. ^∗^*p* < 0.05 and ^∗∗^*p* < 0.01 indicate significant difference and extremely significant difference, respectively.

## 3. Results

### 3.1. MenSC-Derived Exosome Improves BLM-Induced Pulmonary Fibrosis in Mice

**Figure 1:**
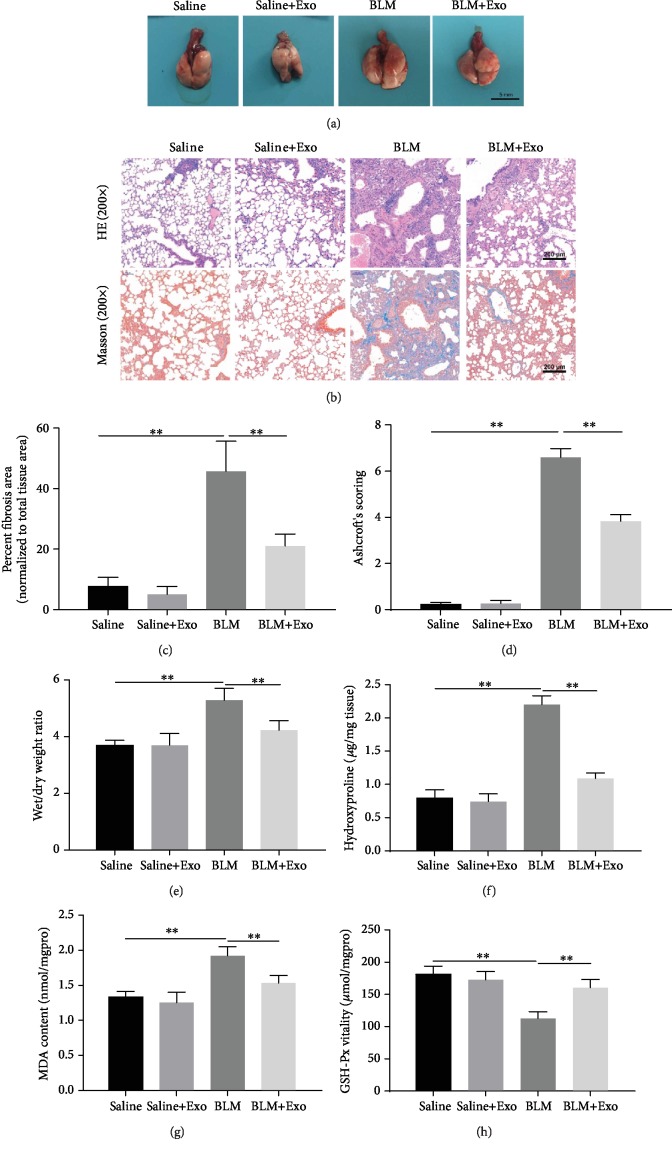
Effects of MenSC-derived exosomes on BLM-induced pulmonary fibrosis in mice. (a) The representative histologic appearance of lung tissue in mice exposed to saline, saline plus exosome, BLM, and BLM plus exosome. Scale bar: 5 mm. (b) HE staining (upper) and Masson staining (lower) of mouse lung tissue in the four groups. Scale bar, 200 *μ*m. (c) Fibrotic area in the four groups was fully quantified (*n* = 6 per group). (d) Ashcroft scores, (e) dry and wet weight ratio of lung tissue, and (f) hydroxyproline detection in the mouse lung tissue of four different groups (*n* = 6 per group). (g) Measurements of malondialdehyde (MDA) content and (h) glutathione peroxidase (GSH-Px) level in mouse lung tissue using detection kits (*n* = 6). Data is shown as the means ± SD. ∗ indicates the difference between saline and BLM or the difference between BLM and BLM plus exosome. ^∗∗^*p* < 0.01.

Many studies have confirmed that stem cell-secreted exosomes contribute to the improvement of lung disease [19, 28]. To explore the role of exosomes on pulmonary fibrosis, MenSCs were firstly collected and isolated from the menstrual blood of female healthy subjects ([Supplementary-material supplementary-material-1]). MenSCs were identified using stem cell positive markers CD44, CD90, and CD105 and negative markers CD34 and CD45 by flow cytometry ([Supplementary-material supplementary-material-1]). The isolated MenSCs also had strong adipogenic and osteogenic differentiation ability by Oil Red O and Alizarin Red staining ([Supplementary-material supplementary-material-1]), which indicates that MenSCs were successfully isolated and used for further research. Next, MenSC-derived exosomes were isolated and verified by transmission electron microscopy, WB, and NTA detection. The obtained exosomes were found to be uniform in particle size averaged between 40 and 150 nm, and the protein levels of TSG101, CD9, and CD63 (positive markers of exosomes) were highly expressed, while calnexin (negative marker of exosomes) showed a low level in MenSC-derived exosomes ([Supplementary-material supplementary-material-1]). After that, the BLM-induced mice model with pulmonary fibrosis was employed to determine the effect of MenSC-secreted exosomes on its pathogenic process. The results showed that the predominant gross lesions of the lung were pale, mottled, and swollen, which was relieved by the administration of MenSC-secreted exosomes (Figure 1(a)). Compared with the control group, the lung alveolar and alveolar wall structures of the BLM group were unclear and there was a large amount of hyperplastic connective tissue (Figure 1(b)). There also showed a larger amount of blue collagen deposition in the BLM group (Figure 1(b)). In addition, the fibrotic area and fibrosis score were significantly increased (Figures 1(c) and 1(d)), and the dry and wet specific gravity were adjusted in the BLM model group (Figure 1(e)). In comparison with the BLM group, there presented lessening symptoms of pulmonary fibrosis, the reduction of blue collagen deposition, and the decline of dry and wet specific gravity in the model mice treated with exosomes (Figures 1(b)–1(e)). The levels of hydroxyproline, malondialdehyde, and glutathione peroxidase in mouse lung tissues were further examined. It could be seen that BLM caused a significant increase in hydroxyproline and malondialdehyde (MDA) levels in the lung tissue of mice and a significant decrease in glutathione peroxidase (GSH-Px) levels (Figures 1(f)–1(h)). Once treated with MenSC-derived exosome, the increase of hydroxyproline and malondialdehyde and the decline of glutathione peroxidase levels were significantly rescued (Figures 1(f) and 1(h)). The above results indicate that exosomes from MenSCs have a certain protective effect on pulmonary fibrosis.

### 3.2. MenSC-Derived Exosomes Improved BLM-Induced Alveolar Epithelial Cell Damage

**Figure 2:**
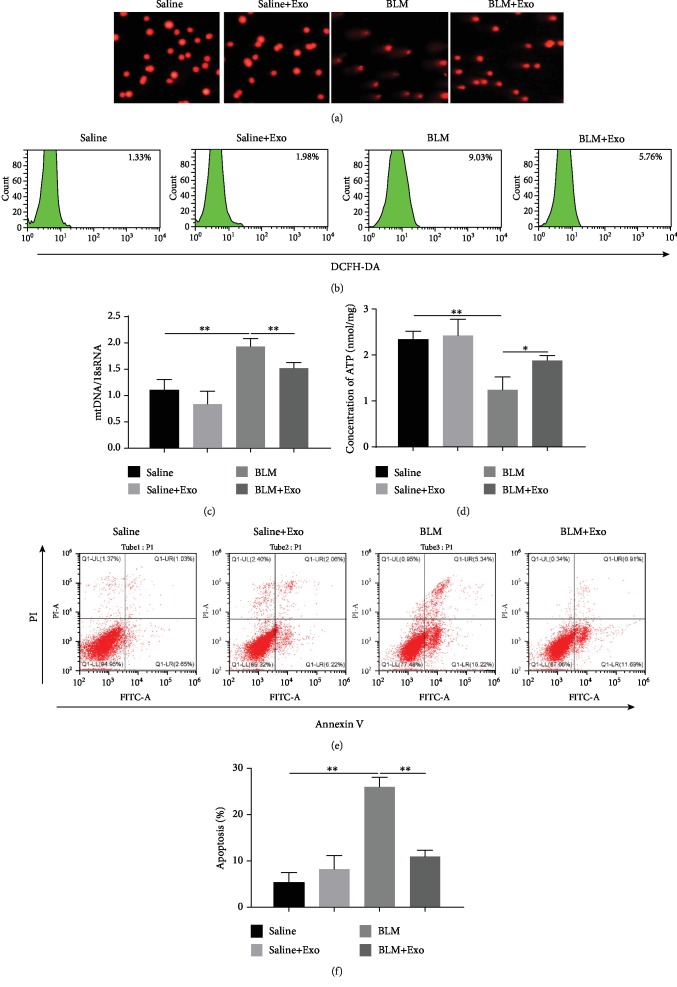
Effect of MenSC-derived exosomes on mtDNA damage in primary alveolar epithelial cells. (a) Primary alveolar epithelial cells were isolated from the four mouse groups. Then, DNA damage was measured by comet assay. (b) Single cells from lung tissue were incubated with 1 mM DCFH-DA at 37°C for 30 min, and reactive oxygen species (ROS) were tested by flow cytometry using DCFH-DA ROS fluorescence probe. (c) Cellular mtDNA/18sRNA and (d) intracellular ATP levels were determined by their detection kits according to the manufacturer's instruction (*n* = 6). (e) Detection of cell apoptosis in the four groups as determined by flow cytometry using an Annexin V-FITC kit. (f) Quantitative statistics of the level of apoptosis in panel (e) (*n* = 3). Data is shown as the means ± SD. ∗ indicates the difference between saline and BLM or the difference between BLM and BLM plus exosome. ^∗^*p* < 0.05 and ^∗∗^*p* < 0.01.

Next, primary alveolar epithelial cells were selected to discover the reason of MenSC-derived exosome-mediated protection on pulmonary fibrosis. In the comet assay, we found that exosome addition protected the alveolar epithelial cell from BLM-mediated DNA damage and the increase in the number of comet tail moment (Figure 2(a)). After treatment with BLM, the levels of reactive oxygen species (ROS) and the ratio of mtDNA/18sRNA in the cells were notably increased, while the ATP level decreased sharply (Figures 2(b)–2(d) and [Supplementary-material supplementary-material-1]). Additionally, BLM exposure also induced the increase of the cell apoptosis rate (Figures 2(e) and 2(f)). However, once treated with MenSCs-Exo, BLM induced the upregulation of ROS activity, mtDNA/18sRNA ratio, and cell apoptosis, and the decrease of ATP levels were robustly rescued (Figures 2(b)–2(f) and [Supplementary-material supplementary-material-1]). Given that ROS accumulation facilitates the upregulation of mtDNA in the alveolar epithelial cell, resulting in fibrosis of the fibroblast [13], the protection role of MenSC-derived exosomes on alveolar epithelial cell damage is possibly through inhibiting ROS/mtDNA/fibrosis signaling cascades.

### 3.3. Exosomes Mediate the Shuttling of Let-7 into Alveolar Epithelial Cells

**Figure 3:**
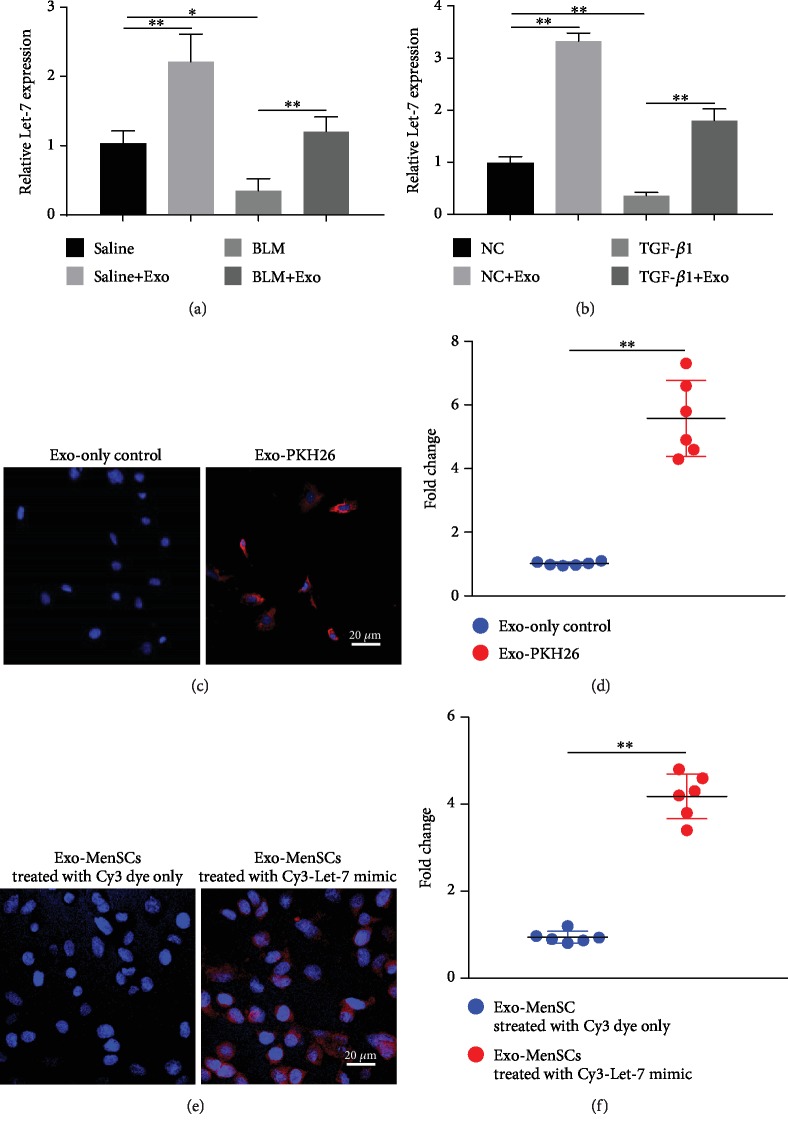
The shuttling of exosomal Let-7 between MenSCs and recipient cells. (a) Total RNA was first extracted from mice lung tissues and detection of Let-7 content in the four groups as measured by qRT-PCR (*n* = 6). (b) MLE-12 cells were exposed to TGF-*β*1, exosome, or TGF-*β*1 plus exosome for 48 h. Then, Let-7 content in MLE-12 cells as evaluated by qRT-PCR (*n* = 3). (c) MenSC-derived exosome was labeled with PKH26 and exposed to MLE-12 cells. Fluorescence intensity in MLE-12 cells coincubated with MenSC-derived exosomes labeled with PKH26 or not was performed by immunofluorescence. (d) Relative quantitative of the fluorescence content of the PKH26-labeled exosomes in MLE-12 cells (*n* = 6). (e) Fluorescence expression analysis of Cy3-labeled Let-7 mimic in MLE-12 cells treated with MenSC-derived exosomes (cells were firstly transfected with Cy3 and Cy3-labeled Let-7 mimic). (f) Quantitative statistics of the fluorescence content of Cy3-labeled Let-7 mimic in MLE-12 cells (*n* = 6). Data is shown as the means ± SD. ^∗^*p* < 0.05 and ^∗∗^*p* < 0.01.

Exosomal miRNAs are the main functional unit of exosomes. In our study, the expression of Let-7 in lung tissue of BLM-exposed mice was significantly lower than that of the saline group (Figure 3(a)). In comparison to the saline group, exosome treatment alone promoted the level of Let-7 in lung tissue and in addition also blunted the inhibition role of BLM on Let-7 expression in the BLM plus exosome group (Figure 3(a)). Similarly, exosome exposure also restored the TGF-*β*1-induced decrease of the Let-7 expression in MLE-12 cells (Figure 3(b)). To further confirm whether Let-7 is a communication bridge between exosomes and alveolar epithelial cells, exosomes were firstly labeled with PKH26. After incubation with PKH26-labeled exosomes, there was an amount of red fluorescence in MLE-12 cells, which indicated that MenSCs-Exo were able to enter the recipient alveolar epithelial cells (Figure 3(c)). In order to verify the uptake of Let-7 derived from MenSCs-Exo in recipient alveolar epithelial cells, MenSCs were transfected with Cy3-labeled Let-7 mimic, and MenSC-derived exosomes were isolated for incubation with MLE-12 cells. As shown in Figure 3(d), red fluorescence appeared in the recipient cells after incubation for 48 h, implying that MenSCs-Exo mediate the shuttling of Let-7 into recipient cells (alveolar epithelial cells) (Figures 3(d) and 3(e)).

### 3.4. Let-7 Inhibition Blocks Exosome-Launched Improvement of Alveolar Epithelial Cell Damage and Pulmonary Fibrosis

**Figure 4:**
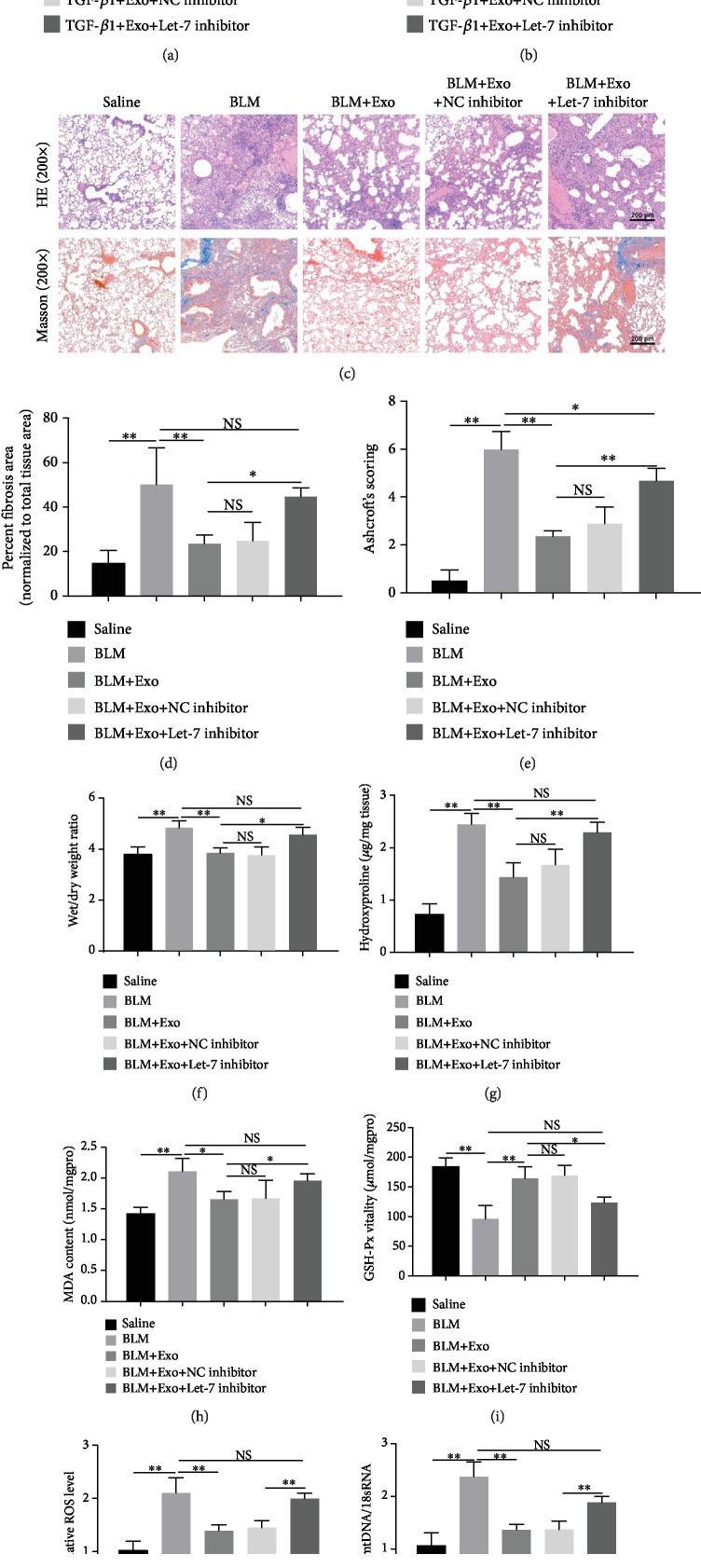
Effect of Let-7 modification on alveolar epithelial cell injury and pulmonary fibrosis. (a) Cells were firstly transfected with Let-7 NC/inhibitor for 24 h. Then, the relative level of ROS in MLE-12 cells exposed to TGF-*β*1, TGF-*β*1 plus exosome, and three combinations of TGF-*β*1, exosome (48 h), and Let-7 inhibitor or Let-7 NC inhibitor was determined by flow cytometry using DCFH-DA ROS fluorescence probe (*n* = 3). (b) mtDNA damage in MLE-12 cells of each group as measured by PCR (*n* = 3). (c) HE staining and Masson staining were performed in mice exposed to saline, BLM, BLM plus exosome, BLM plus exosome and negative control of Let-7, and BLM plus exosome and Let-7 inhibitor (*n* = 6). Scale bar, 200 *μ*m. (d) Fibrotic area in the five groups was fully quantified (*n* = 6 per group). (e) Ashcroft scores, (f) dry and wet weight ratio of lung tissue, and (g) hydroxyproline detection in the mouse lung tissue of four different groups (*n* = 6 per group). (h) Measurements of malondialdehyde (MDA) content and (i) glutathione peroxidase (GSH-Px) level in mouse lung tissue using their corresponding detection kits. (j) The expression level of ROS in the lung tissue of each group as assessed by flow cytometry using a DCFH-DA assay. (k) Statistics of mtDNA damage in lung tissues of mice in each group (*n* = 6 per group). Data is shown as the means ± SD. ^∗^*p* < 0.05 and ^∗∗^*p* < 0.01.

To demonstrate the regulatory role of Let-7 in the process of MenSC-derived exosome-mediated improvement of fibrosis, we proceeded with gene modification of Let-7 by using its inhibitor *in vitro* and *in vivo*. In MLE-12 cells, Let-7 inhibitor administration obviously impeded the protective role of exosomes on the ROS level and mtDNA/18sRNA ratio which were induced under the condition of TGF-*β*1 (Figures 4(a) and 4(b)). In the *in vivo* assay, similar to the above results, exosome addition alleviated BLM-induced a large amount of connective tissue hyperplasis and blue collagen deposition in pulmonary tissue. However, Let-7 inhibition reversed the protective role of exosomes on the degree of fibrosis and blue collagen deposition (Figure 4(c)). Along with these changes, MenSC-Exo management-mediated improvement on index parameters of pulmonary fibrosis in BLM model mice, including the fibrotic area, fibrosis score, wet/dry weight ratio, hydroxyproline, MDA, and GSH-Px vitality, was blunted notably by the interference of the Let-7 inhibitor (Figures 4(d)–4(i)). Additionally, exosome-mediated recovery in BLM-mediated increase of ROS and the ratio of mtDNA/18sRNA in mouse lung tissue were counteracted by Let-7 inhibition (Figures 4(j) and 4(k)). However, Let-7 inhibitor addition did not completely reverse the protective role of exosomes on pulmonary fibrosis indexes, including the ROS level and fibrosis in vitro and in vivo (Figures 4(a) and 4(e), the second row vs. the fifth row). These data suggested that the exosome-mediated improvement role on pulmonary fibrosis damage did not only depend on Let 7; there might be other functional contents in the MenSCs-Exo. Collectively, the downregulation of Let-7 significantly inhibited the improvement of exosomes on lung fibrosis and mtDNA damage in lung tissue and alveolar epithelial cell.

### 3.5. Exosomal Let-7 Targets LOX1 in Alveolar Epithelial Cells

**Figure 5:**
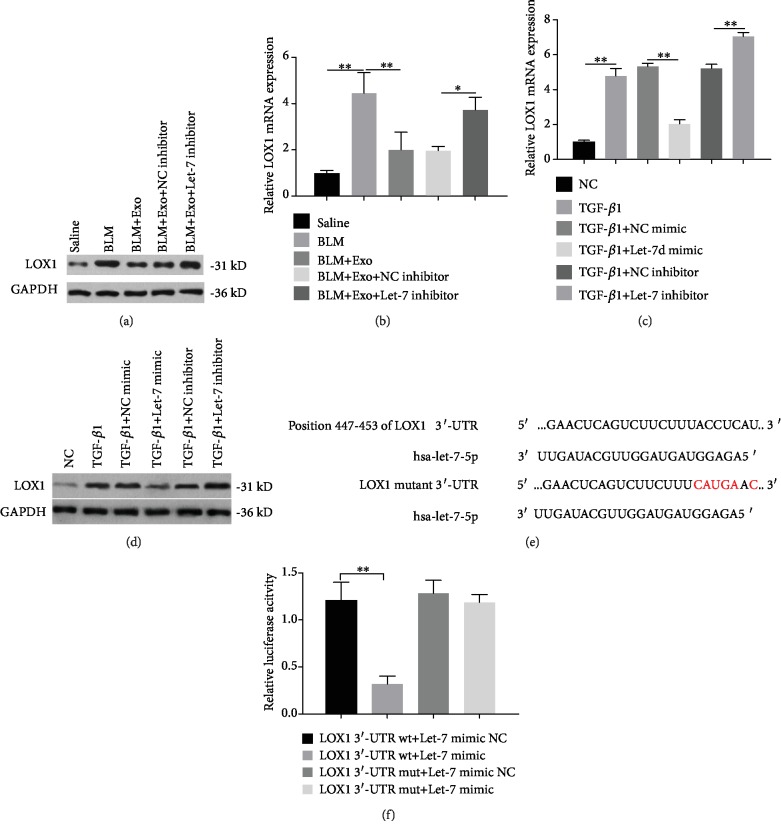
The targeting regulatory effect of Let-7 on LOX1. (a) Total protein was extracted from the lung tissue of saline, BLM, BLM plus exosome, BLM plus exosome and negative control of Let-7, and BLM plus exosome and Let-7 inhibitor groups. Then, the expression of LOX1 protein was detected by western blotting. GAPDH was served as the internal control. (b) The transcription level of LOX1 in the mouse lung tissue of each group was determined by qRT-PCR (*n* = 6). (c) MLE-12 cells were exposed to TGF-*β*1, TGF-*β*1 plus NC mimic, TGF-*β*1 plus Let-7 mimic, TGF-*β*1 plus NC inhibitor, and TGF-*β*1 plus Let-7 inhibitor. Then, the transcription level of LOX1 in these cells was determined by qRT-PCR (*n* = 3). (d) Protein expression of LOX1 was detected by WB in the above cells. (e) Schematic diagram of the binding site of LOX1 3′-UTR to Let-7-5p and the mutation of LOX1 3′-UTR. (f) Luciferase activity of LOX1 was measured in cells transfected with Let-7 mimic and LOX1 3′-UTR plasmids (*n* = 3). Data is shown as the means ± SD. ^∗^*p* < 0.05 and ^∗∗^*p* < 0.01.

A previous study has indicated that endothelial Let-7 targets lectin-like oxidized low-density lipoprotein scavenger receptor-1 (LOX1) and represses its expression in the vascular smooth muscle cell [29]. In the present study, compared with the control group, LOX1 protein and transcriptional expression levels were increased in the lung tissue of the BLM group (Figures 5(a) and 5(b)). Once treated with exosomes, BLM-mediated enhancement of LOX1 levels was observably reduced, in which changes were robustly repressed by the addition of a Let-7 inhibitor (Figures 5(a) and 5(b)). In the *in vitro* model, the protein and transcript levels of LOX1 were also upregulated in MLE-12 cells exposed to TGF-*β*1, whereas addition of the Let-7 mimic decreased, and addition of the Let-7 inhibitor promoted, the TGF-*β*1-induced upregulation of LOX1 (Figures 5(c) and 5(d)). Further analysis found that there was a potential binding site of Let-7 in LOX1 the 3′-UTR region based on TargetScan (Figure 5(e)). Based on the dual luciferase reporter gene assay, we observed that the Let-7 mimic had a direct inhibition effect on the luciferase ability of wild-type LOX1 3′-UTR but had no effect on that of mutated LOX1 3′-UTR (Figure 5(f)). The above results indicate that exogenous Let-7 transported via exosomes may function like an endogenous miRNA targeting LOX1 in alveolar epithelial cells.

### 3.6. LOX1 Mediates Apoptosis of Alveolar Epithelial Cells by Upregulating mtDNA Damage and Downstream NLRP3 Activity

**Figure 6:**
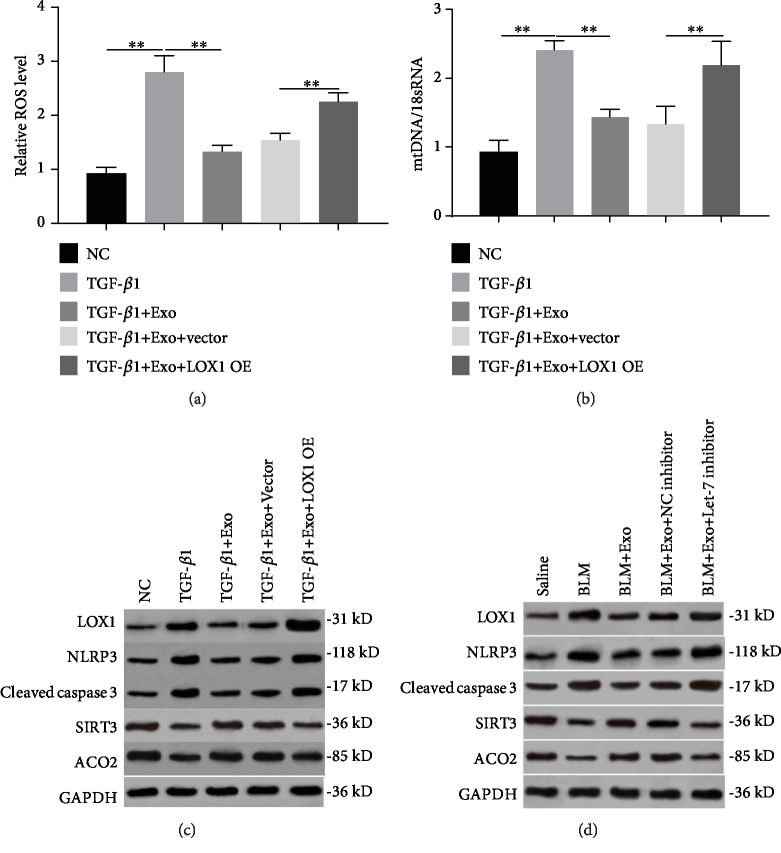
The regulation mechanism of LOX1 on alveolar epithelial cell apoptosis and fibrosis. (a) Cells were transfected with a LOX1 overexpression plasmid and control vector. ROS levels were determined by DCFH-DA assay in MLE-12 cells treated by TGF-*β*1, TGF-*β*1 plus exosome, TGF-*β*1 plus exosome and vector, and TGF-*β*1 plus exosome and LOX1 overexpression plasmid (*n* = 3). (b) Detection of cellular mtDNA/18sRNA in each of the above cell groups (*n* = 3). (c) The expression of LOX1, caspase 3, mtDNA damage markers SIRT3 and ACO2, and NLRP3 was measured by western blotting in each of the above cell groups. (d) The expression of the same signal cascades including LOX1, caspase 3, SIRT3, ACO2, and NLRP3 was confirmed in an animal model with pulmonary fibrosis by western blot assay. Data is shown as the means ± SD, *n* ≥ 3. ^∗∗^*p* < 0.01.

Then, we employed the overexpressed LOX1 plasmid to demonstrate its role on alveolar epithelial cell apoptosis. Similar to the effect of the Let-7 inhibitor, forced expression of LOX1 also blocked the improvement role of exosomes on TGF-*β*1-mediated ROS activation and mitochondrial DNA damage (Figures 6(a) and 6(b)). Western blot results further confirmed that TGF-*β*1 stimulation notably promoted the expression of LOX1 and downstream NLRP3 activity, leading to the activation of caspase 3, while inhibited the expression of mitochondrial DNA injury markers including SIRT3 and ACO2 (Figure 6(c) and [Supplementary-material supplementary-material-1], the second row vs. the first row). Under the exosome condition, the increase of LOX1, NLRP3, and cleaved caspase 3 and the decrease of SIRT3 and ACO2 were reversed compared with the TGF-*β*1-exposed group (Figure 6(c) and [Supplementary-material supplementary-material-1], the third row vs. the second row). However, LOX1 overexpression obviously promoted the signal cascades of LOX1/NLRP3/caspase 3 and the reduction of SIRT3 and ACO2 levels. Also, upregulation of LOX1 weakened the improvement effect of exosomes (Figure 6(c), the fifth row vs. third and fourth rows). Additionally, as shown in Figure 6(d), the activation of the LOX1/NLRP3/caspase 3 pathway and the decline of SIRT3 and ACO2 protein expression were found in the BLM-treated mice (Figure 6(d) and [Supplementary-material supplementary-material-1]). Exosome addition was able to reverse the changes in the protein expression that were described above (Figure 6(d) and [Supplementary-material supplementary-material-1]). However, the Let-7 inhibitor treatment neutralized the intervention effect of exosomes on LOX1/NLRP3/caspase 3 and mtDNA damage (Figure 6(d) and [Supplementary-material supplementary-material-1]). These results demonstrate that LOX1 can promote the apoptosis of alveolar epithelial cells via affecting mtDNA processes and NLRP3 activities and reversing the protective process of exocyoids on pulmonary fibrosis.

## 4. Discussion

Studies have shown that the severity of lung fibrosis depends on the proliferation rate of alveolar epithelial cells (AEC) and the repair of epithelial damage [30, 31]. Increased apoptosis of type II alveolar epithelial cells (AECIIs) was observed in proliferating epithelial cells covering fibroblastic foci [32]. Blockade of the apoptotic pathway attenuates the degree of fibrosis in the pulmonary fibrosis bleomycin model [33]. Inappropriate apoptosis in AEC promotes fibrogenesis by eliminating its “antifibrotic” function, which includes inhibition of lung fibroblast proliferation and fibrinolysis [34, 35]. In this study, MenSC-derived exosomes were able to improve pulmonary fibrosis by regulating alveolar epithelial cell apoptosis. This finding provides new insights and methods for improving pulmonary fibrosis through targeting alveolar epithelial cell apoptosis.

Once injected with MenSCs, it will be primarily captured and adsorbed in the lungs. Early treatment with MenSCs can prevent inflammation, inhibit the subsequent development of fibrosis, and protect alveolar epithelial cells from damage [25]. In AEC exposed to asbestos, mitochondrial ROS production is required for asbestos-induced mtDNA damage and apoptosis [36]. In mice lacking an oxidant-induced DNA repair enzyme, the mitochondrial dysfunction, mitochondrial DNA damage, and cell apoptosis also increased significantly, resulting in the process of pulmonary fibrosis [37–39]. This suggests that mitochondrial ROS production, mtDNA damage, and apoptosis play an important role in promoting pulmonary fibrosis. In this study, MenSC-Exo treatment had a remission role on BLM-induced pulmonary fibrosis *in vivo* by inhibiting several fibrosis indices. In *in vitro* assays, we found that the BLM-evoked increase of cellular ROS, mtDNA damage, and apoptosis was robustly reduced by the addition of exosomes. The above results imply that the MenSC-secreted exosome might have a protective effect on fibrosis and alveolar epithelial cell damage through affecting ROS production and mtDNA damage.

Stem cell-derived exosomal miRNAs are reported to be involved in fibrosis and stem cell pluripotency, including miR-23a and miR-203a which inhibit Smad 2 and Smad 3, respectively, contributing to TGF-*β*-mediated inhibition on epithelial-mesenchymal transition [40, 41]. Accumulating evidence has indicated that miRNAs function as a key regulator in the development of organ fibrosis. miR-34a is able to induce lung fibroblast senescence and inhibit fibroblast proliferation [42]. miR-150 and miR-194 inhibit the activation of hepatic stellate cells by inhibiting the expression of c-MYB and RAC1, leading to the reduction of collagen deposition in liver fibrosis [43]. One of the most abundant antifibrotic miRNAs is miR-27a, and lentiviral delivery of miR-27a-3p has been reported to reduce BLM-induced pulmonary fibrosis by targeting the phenotypic marker of myofibroblasts, alpha-smooth muscle actin (*α*-SMA), and two key Smad transcription factors, Smad2 and Smad4 [44]. In our study, it was found that MenSCs-Exo mediated the transport of Let-7 to alveolar epithelial cells as a key substance during the procedure of MenSC-Exo-mediated improvement of fibrosis and lung epithelial cell damage. The results also showed that inhibition of Let-7 significantly inhibited the protective role of exosomes on lung fibrosis and alveolar epithelial damage *in vitro* and *in vivo*. These findings suggest that exosome mediates the shuttling of Let-7 into alveolar epithelial cells and the enhancement of Let-7 subsequently affects the procedure of ROS production and mtDNA damage in epithelial cells, resulting in epithelial cell injury. Possibly, the entrance of exosomal Let-7 into alveolar epithelial cells not only acts as a protective factor in cell damage but also inhibits the procedure of EMT and subsequent lung fibrosis in mice. However, exosomes can carry more than one miRNA, and there may be other miRNA contents in MenSCs-Exo such as miR-122 and miR-181 that also play a protective role in fibrosis [22, 45, 46]. Therefore, future studies must explore the contribution of other exosomal miRNAs to the antifibrosis effect and make MenSCs-Exo develop into the therapeutic agents for IPF.

LOX1, a receptor for oxidized low-density lipoprotein (oxLDL) in endothelial cells, is upregulated in many inflammation-related pathophysiological events such as diabetic pulmonary fibrosis [29, 47]. Excessive production of ROS is able to induce the expression of LOX1, accompanied by mtDNA damage and autophagy activation [48]. It has been reported that LOX1 elimination motivates inflammatory responses as well as mtDNA damage, and mice lacking LOX1 function as the limited role of the activity of the NLRP3 inflammasome in diabetic pulmonary fibrosis [47]. Age-dependent increase in mitochondrial ROS production and NLRP3 inflammasome activation in alveolar macrophages contribute to the development of pulmonary fibrosis [49]. Therefore, LOX1 can affect the activation of the NLRP 3 inflammasome by affecting ROS and mtDNA damage and then induce pulmonary fibrosis. It has been pointed out that LOX1 is the potential target of Let-7a and Let-7b, which inhibit the expression of LOX1 by targeting the position of 310-316 in the region of LOX1 3′-UTR [29]. This study found that Let-7 could target the regulation of LOX1 in lung epithelial cells and exosomes can alter the expression of LOX1 in epithelial cells through shuttling of Let-7, which ultimately affected the downstream mtDNA damage and the activation of the NLRP 3 inflammasome. This study initially explores that LOX1 can promote the apoptosis of alveolar epithelial cells through mtDNA/NLRP3 signal cascades and reverse the protection of lung fibrosis by MenSC-derived exosomal Let-7. However, this was only validated in vitro, and there is a lack of *in vivo* experiments on genetic modification of LOX1 to confirm the protective role of the exosomal Let-7/LOX1 pathway on alveolar epithelial cell damage and lung fibrosis, which should be verified by much deeper research.

## 5. Conclusion

In conclusion, the current study indicates that the exosomes derived from MenSCs could ameliorate pulmonary fibrosis by transporting miRNA Let-7 into the alveolar epithelial cell, which subsequently downregulated ROS levels, LOX1 expression, mtDNA damage, and inflammatory body NLRP3 activation. These findings suggest that exosomes from MenSC-based therapies could be a promising strategy for treating IPF.

## Figures and Tables

**Table 1:** Primer sequences of qRT-PCR.

Gene	Primer sequence
hsa-let-7-5p_1sl	GGTTGTGGTTGGTTGGTTTGTATACCACAACCAACTAT
hsa-let-7-5p_1F	AGGGTGAGGTAGTAGGTTGT
hsa-let-7-5p_1R	GTTGTGGTTGGTTGGTTTGT
LOX1_1F	AACAAACTAAGCCAGGTATGC
LOX1_1R	AGAGTGGGTGGAAAGGAAA
GAPDH_1F	CCTTCCGTGTCCCCACT
GAPDH_1R	GCCTGCTTCACCACCTTC
U6_1F	CTCGCTTCGGCAGCACA
U6_1R	AACGCTTCACGAATTTGCGT

## Data Availability

The data used to support the findings of this study are included within the article.

## Supplementary Materials

Supplementary Materials Figure S1: identification of prepared MenSCs. (A) MenSC morphology (third generation) was observed by optical microscope. (B) Detection of CD105, CD90, CD44, CD45, and CD34 expression in MenSCs by flow cytometry. (C) Oil Red O and Alizarin Red staining for detecting the adipogenic osteogenic measurement differentiation abilities of MenSCs. Figure S2: verification of MenSC-derived exosomes. (A) Electron microscopy results (left) and video screenshots from the particle matrix detection (right) of MenSC-derived exosomes. (B) Expressions of TSG101, CD9, CD63, and calnexin were determined by WB in MenSC-derived exosomes. (C) Concentration-particle size distribution of exosomes from MenSCs by NTA detection. Figure S3: relative activity of ROS in lung tissue. Quantitative analysis of the ROS level via evaluating the fluorescence intensity. ^∗∗^*p* < 0.01. Figure S4: relative expression of apoptosis- and mtDNA damage-related signal cascades. Quantitative analysis of LOX1/NLRP3/caspase 3 and mtDNA damage markers SIRT3 and ACO2 in MLE-12 cells (A) and in mice lung tissue (B). ^∗^*p* < 0.05 and ^∗∗^*p* < 0.01.
