# Supplementary material for: Exosomal miRNA Let-7 from Menstrual Blood-Derived Endometrial Stem Cells Alleviates Pulmonary Fibrosis through Regulating Mitochondrial DNA Damage
Source: Oxid Med Cell Longev. 2019 Dec 16;2019:4506303. doi: 10.1155/2019/4506303 (PMC6948326; doi:10.1155/2019/4506303)
Supplement: Supplementary Materials — Figure S1: identification of prepared MenSCs. (A) MenSC morphology (third generation) was observed by optical microscope. (B) Detection of CD105, CD90, CD44, CD45, and CD34 expression in MenSCs by flow cytometry. (C) Oil Red O and Alizarin Red staining for detecting the adipogenic osteogenic measurement differentiation abilities of MenSCs. Figure S2: verification of MenSC-derived exosomes. (A) Electron microscopy results (left) and video screenshots from the particle matrix detection (right) of MenSC-derived exosomes. (B) Expressions of TSG101, CD9, CD63, and calnexin were determined by WB in MenSC-derived exosomes. (C) Concentration-particle size distribution of exosomes from MenSCs by NTA detection. Figure S3: relative activity of ROS in lung tissue. Quantitative analysis of the ROS level via evaluating the fluorescence intensity. ∗∗p < 0.01. Figure S4: relative expression of apoptosis- and mtDNA damage-related signal cascades. Quantitative analysis of LOX1/NLRP3/caspase 3 and mtDNA damage markers SIRT3 and ACO2 in MLE-12 cells (A) and in mice lung tissue (B). ∗p < 0.05 and ∗∗p < 0.01. [file 4506303.f1.pdf]

Fig. S1

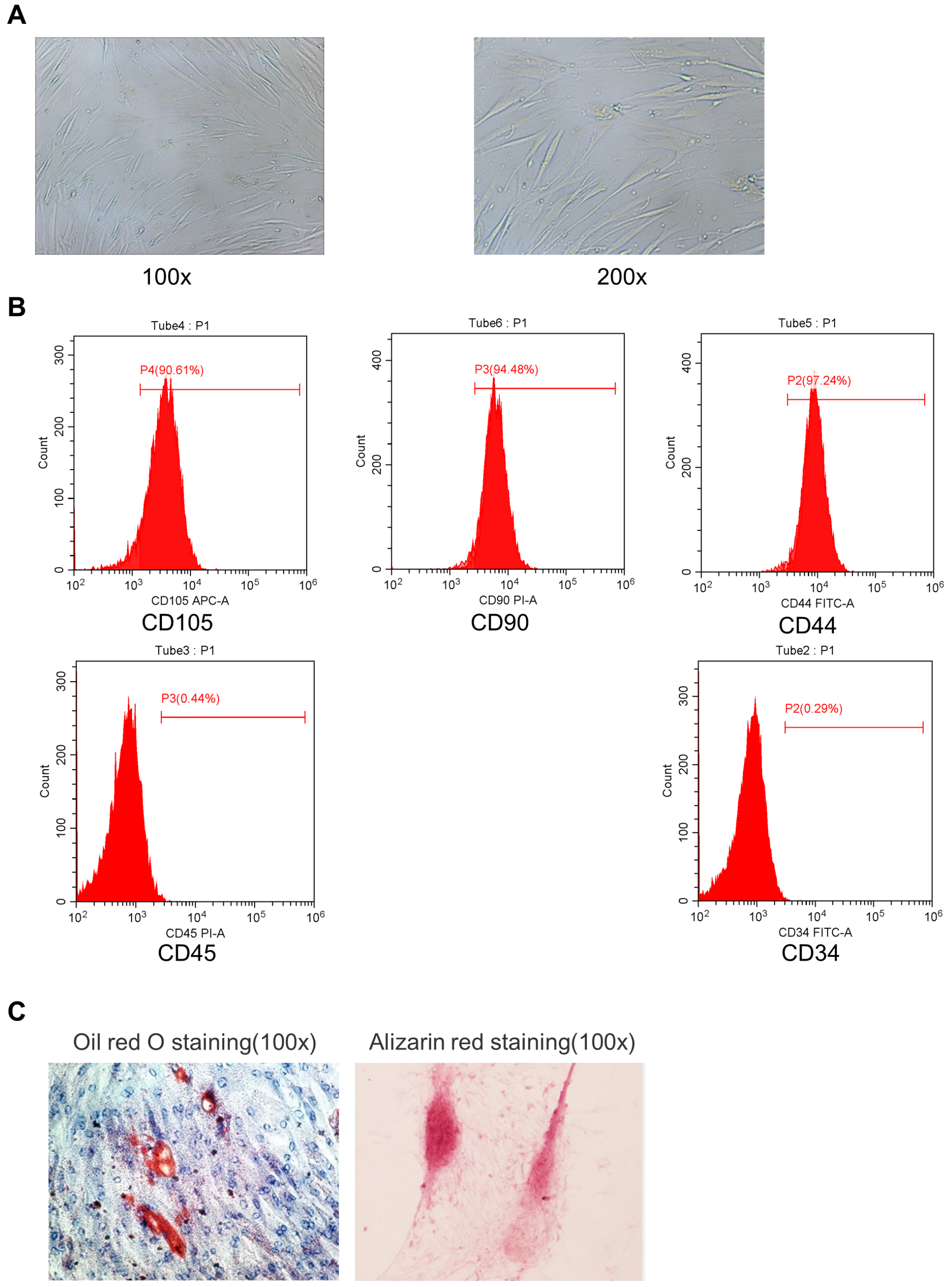

Figure S1. Identification of prepared MenSCs. (A) MenSCs morphology (third generation) were observed by optical microscope. **(B)** Detection of CD105, CD90, CD44, CD45, CD34 expression in MenSCs by flow cytometry. **(C)** Oil red O and Alizarin Red staining for detecting the adipogenic osteogenic measurement differentiation abilities of MenSCs.

Fig. S2

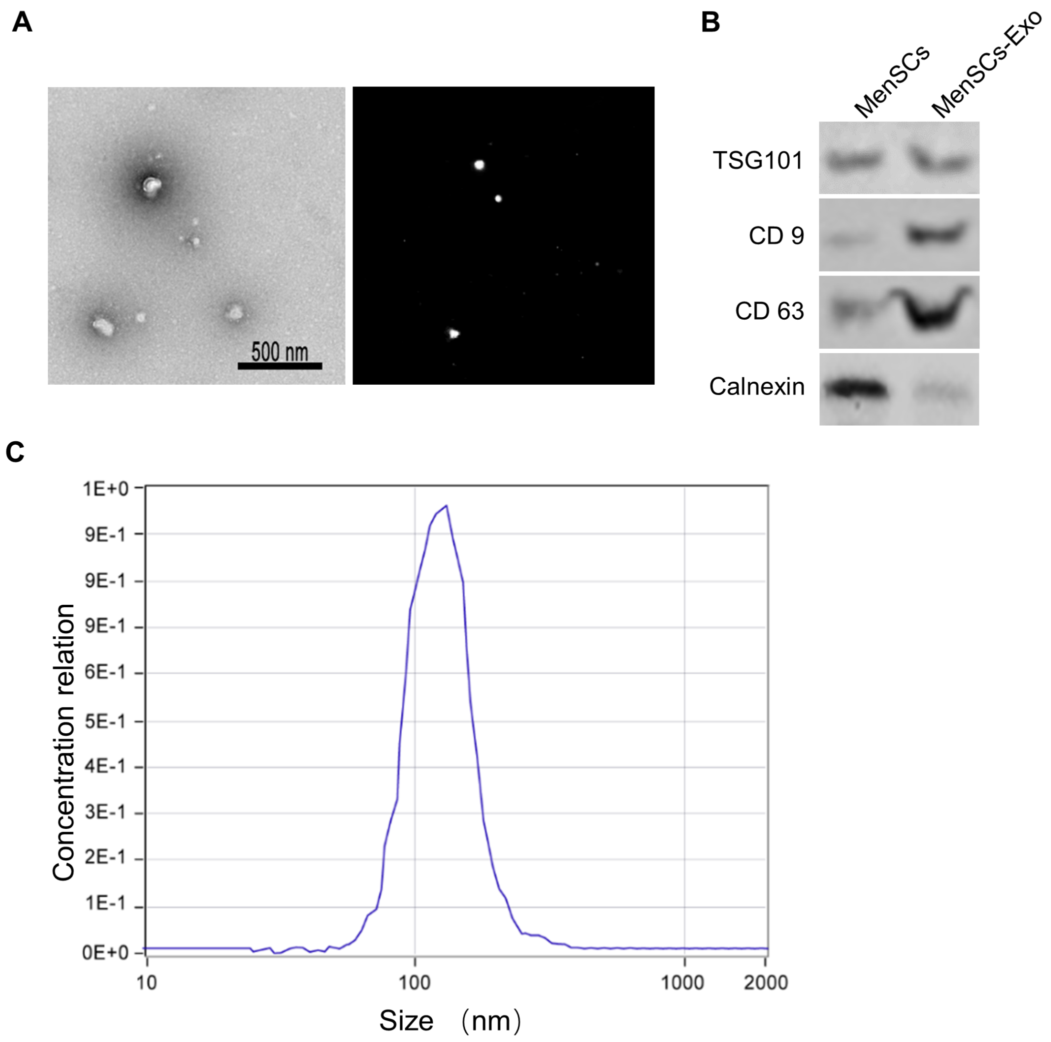

Figure S2. Verification of MenSCs-derived exosome. (A) Electron microscopy results (left) and video screenshots from particle metrix detection (right) of MenSCs-derived exosomes. **(B)** Expression of TSG101, CD9, CD63 and Calnexin were determined by WB in MenSCs-derived exosome. **(C)** Concentration-particle size distribution of exosomes from MenSCs by NTA dectetion.

Fig. S3

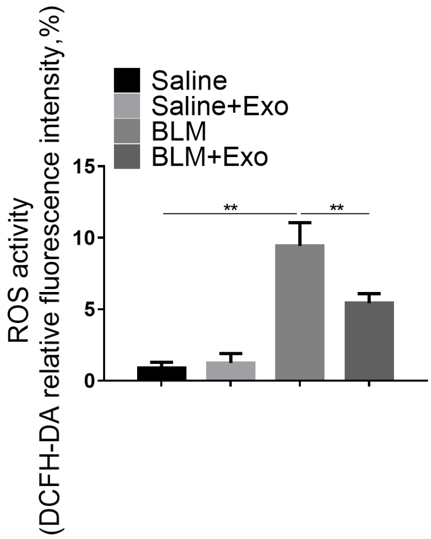

Figure S3. Relative activity of ROS in lung tissue. Quantitative analysis of the ROS level via evaluating the fluorescence intensity. ***p*<0.01.

Fig. S4

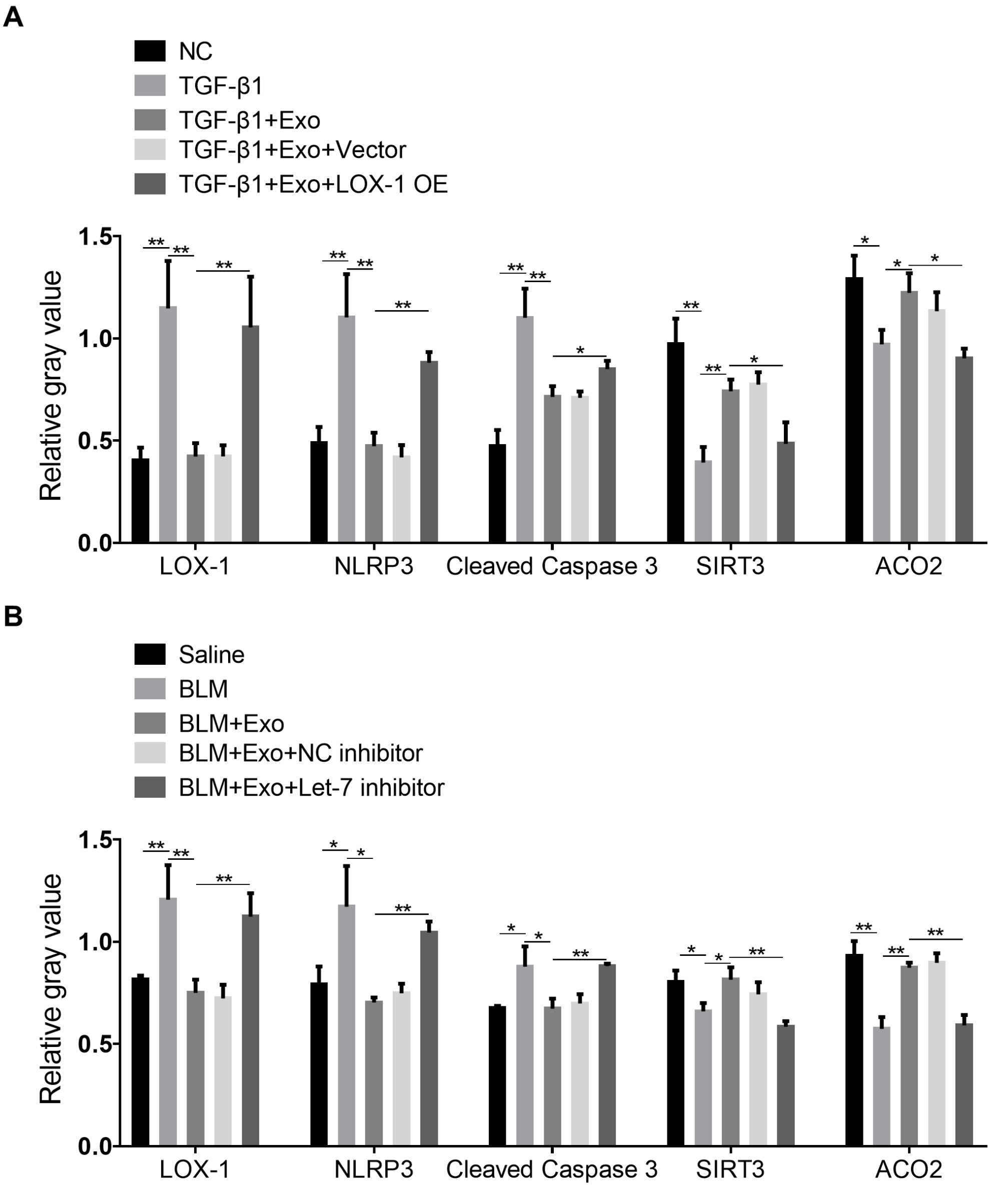

Figure S4. Relative expression of apoptosis- and mtDNA damage-related signal cascades. Quantitative analysis of LOX1/NLRP3/caspase 3 and mtDNA damage markers SIRT3 and ACO2 in MLE-12 cells **(A)** and in mice lung tissue **(B)**. *p<0.05, **p<0.01.
